# Greater heel-rise endurance is related to better gait biomechanics in patients surgically treated for chronic Achilles tendon rupture

**DOI:** 10.1007/s00167-022-06987-4

**Published:** 2022-05-20

**Authors:** Anna Nordenholm, Eric Hamrin Senorski, Katarina Nilsson Helander, Michael Möller, Roland Zügner

**Affiliations:** 1 Department of Health and Rehabilitation, Institute of Neuroscience and Physiology, Sahlgrenska Academy, University of Gothenburg, Medicinaregatan 11-13, 41390 Gothenburg, Sweden; 2 Department of Orthopaedics, Institute of Clinical Sciences, Sahlgrenska Academy, University of Gothenburg, Gothenburg, Sweden

**Keywords:** Chronic Achilles tendon rupture, Calf muscle endurance, Achilles tendon resting angle, Tendon length, Gait analysis

## Abstract

**Purpose:**

To determine the relationships among calf muscle function, tendon length and gait biomechanics in patients surgically treated for chronic Achilles tendon rupture.

**Methods:**

Twenty-one patients with chronic Achilles tendon rupture (mean age 62 ± 13 years) were evaluated by heel-rise endurance test, Achilles Tendon Resting Angle (ATRA), ultrasound measurement of tendon length and three-dimensional gait analysis. A bivariate two-sided correlation test was performed on all variables in all patients.

**Results:**

Better performance across all parameters of the heel-rise endurance test correlated with faster walking speed (*r* = 0.52–0.55), greater peak ankle power (*r* = 0.56–0.64), shorter stance phase (*r* = −0.52 to −0.76) and less peak ankle dorsiflexion angle (*r* = −0.49 to −0.64) during gait. Greater ATRA correlated with longer stance time (*r* = 0.47), greater peak ankle dorsiflexion angle (*r* = 0.48), less heel-rise repetitions (*r* = −0.52) and less heel-rise total work LSI (*r* = −0.44 to −0.59).

**Conclusion:**

Greater calf muscle endurance, especially heel-rise total work, is moderately correlated (*r* = 0.49–0.76) to better ankle biomechanics during gait in patients surgically treated for CATR. The heel-rise endurance test may be a clinical proxy for power development in the ankle joint during gait.

**Level of evidence:**

IV.

## Introduction

Up to 25% of all Achilles tendon ruptures (ATR) have a delayed diagnosis, either due to a misdiagnosis or because of patient delay [1, 2]. When the diagnosis and adequate treatment are delayed for more than 4 weeks after the injury, an ATR is defined as chronic (CATR) [2, 3]. Immediate functional deficits occur when the Achilles tendon ruptures, resulting in inability to perform a single heel-rise as well as alterations in gait and with impaired balance ability [2–4]. The current consensus on treatment after CATR is that surgical repair is preferred to restore tendon length, improve calf muscle function, balance and gait [2, 4–6].

Regardless of treatment, long-term functional deficits such as calf muscle weakness, tendon elongation, and gait abnormalities are common after an acute ATR [7–11]. Similar functional deficits have also been reported after surgical treatment of CATR [12, 13]. These sub-groups of patients are, however, to date, less studied [14–16]. Increased dorsal flexion and decreased plantar flexion range of movement (ROM), as well as impaired peak plantar flexion power on the injured side during gait, have been reported after an acute ATR [8, 10, 17]. However, this has not yet been studied in patients with CATR. In patients with ATR, associations between different functional outcomes have been reported. Brorsson et al. [7] have previously reported that lower heel-rise height correlates with lower eccentric and concentric plantar flexion power during walking, jogging and jumping in patients with ATR when evaluated a mean of 6 years after injury. In turn, greater tendon elongation has also been correlated with lower heel-rise height [18, 19].

The aim of this study was to determine the relationships among calf muscle function, tendon length and gait biomechanics in patients surgically treated for CATR. It was hypothesized that better calf muscle function would correlate with more advantageous gait biomechanics.

## Materials and methods

Ethical approval was obtained from the Swedish Ethical Review Authority (reference number 554-15). Patients with CATR who presented at Sahlgrenska University Hospital and Kungsbacka Hospital in 2014 to 2016 and scheduled for surgical treatment were randomly invited to participate in the study. The inclusion criteria were patients with a unilateral CATR, defined as an ATR that had been left untreated for at least four weeks, who were scheduled for surgical intervention. The first 24 patients who agreed to participate were included in the project. All of them underwent both pre- and 1-year postoperative gait analysis, but solely the data on the postoperative gait analysis were included in the present study. Of them, 22 patients were also evaluated with muscle function tests, clinical measurements of the tendon length with use of ultrasound and patient-reported outcome measures 1-year postoperative. The inclusion criteria in the present study were patients who had undergone both postoperative gait analysis and muscle function tests and clinical tendon measurements with ultrasound. During the evaluations, one patient was excluded due to incorrect inclusion in the study, i.e. too short a time had passed since the ATR. In total, 21 patients with a CATR were finally included in the present study (Table 1).

**Table 1:** Patient demographics

	Mean (SD) Median (min; max)
Age	62 (13) 67 (31; 82) *n* = 21
Male	*n* = 13 (62%)
Female	*n* = 8 (38%)
Weight (kg)	85 (15) 81 (60; 114) *n* = 21
Height (cm)	173 (9) 171 (157; 190) *n* = 21
BMI (kg/m^2^)	28 (4) 28 (22; 37) *n* = 21

### Surgical technique and postoperative care

Twenty-one patients with CATR were treated with an end-to-end suture and augmentation with a free flap from the gastrocnemius aponeurosis after reconstruction of the Achilles tendon, often including shortening, a surgical technique previously presented by Nilsson Helander et al. [20]. One of the patients also received a suture anchor in the calcaneus due to a distal but still mid-portion location of the ATR. In one patient, a free semitendinosus autograft was used instead due to a greater tendon gap. Surgical treatment was performed between 1 and 36 months after injury (median 7 months). A below-the-knee plaster cast was used for three to five weeks postoperatively, followed by an adjustable lower leg brace (DonJoy ROM Walker). Partial load during gait was allowed from three weeks and was successively increased to full weight-bearing in the brace after six weeks. The brace was removed after a total of eight to nine weeks after surgery and the patient continued physical therapy for individual exercise therapy and load instructions based on the current regional rehabilitation protocol. One patient reported a superficial wound complication.

### Gait analysis

The gait analysis was performed at one year postoperatively using an optical tracking system (OTS) according to a standardized protocol, where the test subjects wore underwear and walked barefoot on the floor, one year postoperatively. A total of 15 spherical markers (ø 12 mm) were attached to the skin of the lower extremities and the pelvis with double-adhesive tape by an experienced examiner (RZ). They were placed according to a skin marker model based on Kit Vaughan, presented in detail by Weidow et al. [21] and validated by Tranberg et al. [22] and Zügner et al. [23, 24]. The skin marker model has good agreement compared with the gold standard, Roentgen stereophotogrammetric analysis (RSA) [22, 24]. Markers were attached to the proximal border of the sacrum, anterior and superior iliac spine, lateral knee joint line, proximal boarder of the patella, tibial tubercle, tuber calcanei, lateral malleolus and between the second and third metatarsals. A modified Coda pelvis was used to define the pelvis segment [25]. The modification consisted of a reduction of the two bilateral markers on the posterior superior iliac spine that were replaced by one marker at the mid-point of the proximal border of the sacrum.

A 16-camera motion capture system with a sampling rate of 240 Hz (Oqus 700+, Qualisys AB, Göteborg, Sweden) together with 4 force plates (Amti Optima OPT400600-HF-2K-CTT) was used for data acquisition. Prior to the gait analysis, a static recording with the test subject standing in an upright position in the calibrated volume aligned to the global coordinate system was performed to scale the subject’s anthropological measurements in relation to the marker positions. The patients were then asked to walk 5–10 times at a self-selected speed through the calibrated volume to familiarize themselves with the test. They then performed 6 gait trials of which the approved trials for each test subject (median 5, range 1–6) were selected for further evaluation. The mean of approved trials for each test subject was used in the analysis to increase the reliability of the testing and in order not to miss valuable data, patients with few preoperative trials were also included. A trial was excluded from the analysis if the patient missed to step on the force plates or due to other technical problems. Prior to any calculations, the marker data obtained from the recordings were filtered using a Butterworth fourth-order filter with a cut-off frequency of 6 Hz. For calculations of spatiotemporal, kinematic and kinetic gait variables, Visual 3D™ software (C-Motion, Inc., Germatown, USA) was used.

The collected spatiotemporal variables that were used for the analysis were speed (m/s) and stance phase (% of total gait cycle relative to swing phase). The kinematic variables included in the analysis were the degree of dorsiflexion during stance phase and the kinetic variable collected in the sagittal plane during the stance phase was power (W/kg) in the ankle joint.

### Calf muscle function and tendon length

The muscle function tests and clinical measurements of tendon length were performed at the same day as the gait analysis, at one year postoperatively, and were led by one experienced physical therapist at the orthopaedic research laboratory. The patients received standardized instructions of the test procedure and warm-up prior to testing. Verbal encouragements were given during the tests and all the patients wore standardized athletic footwear. For the evaluation of calf muscle endurance and jumping performance, the MuscleLab® (Ergotest Technology, Oslo, Norway) measurement system was used.

#### Heel-rise endurance test

To evaluate calf muscle endurance, a single-leg standing heel-rise test was used. The heel-rise endurance test has good test–retest reliability (ICC = 0.78–0.84) and a greater ability to detect differences between injured and uninjured side compared with a test only measuring the number of repetitions performed [26]. Standing on a box with a 10-degree incline and allowed to use balance support with the fingertips on the wall at shoulder height, the patients were told to perform as many repetitions as possible and go as high as possible during each heel-rise, while keeping the knee extended. A metronome was used to keep the pace at 30 heel-rises a minute and the test was finished when the patients were unable to rise above 2 cm or were unable to maintain the pace. The linear encoder unit connected to the MuscleLab® measurement system recorded the number of successful heel-rise repetitions, the average height of the heel-rises (cm) and the computed total work (joule), which is the product of the body weight and the total distance through which the body moves.

#### Ultrasound measurement

The Achilles tendon length was measured bilaterally using extended field of view ultrasonography (Logiq E BT09 Ultrasound; GE Healthcare Sweden AB), a method reported to have excellent test–retest reliability (ICC = 0.90–0.97) and validity (ICC = 0.90) when the length of a healthy Achilles tendon is compared between cadaveric measurements [27, 28]. The distance between the calcaneal osteotendinous junction (OTJ) and the gastrocnemius musculotendinous junction (MTJ) was measured using a wideband array linear probe (4C-RS, 5.0–13.0 MHz). The B-mode at 10 MHz and a depth of 3 cm were used to record the images. Three images of each Achilles tendon were recorded and measured in centimetres with a measurement accuracy of 0.5 cm. The mean value was used for data analysis and results are presented in centimetres. The measured value represents the absolute ultrasound measurement, while the relative measurement is the difference between the ATRA on the injured side and the non-injured side.

#### Achilles tendon resting angle

The Achilles tendon resting angle (ATRA) has previously been reported to have excellent test–retest reliability (ICC = 0.80–0.97) and acceptable construct validity when compared to ultrasound measurement [29, 30]. With the patient in a prone position and the knee passively flexed at 90°, a goniometer with one-degree increments was placed with one arm along the shaft of the fibula aligned with the centre of the fibula head and the other arm aligned with the head of the fifth metatarsal. The rotational centre of the goniometer was placed on the tip of the lateral malleolus and the angle between the arms was used for analysis. The measured value represents the absolute ATRA, while the relative ATRA is the difference between the ATRA on the injured side and the non-injured side [18].

### Statistical analysis

Statistical analyses were performed using IBM SPSS Statistics for Mac, Version 26. Descriptive data were reported as mean (standard deviation) and median (min; max). All variables were not normally distributed; therefore, the non-parametric Spearman’s rho was used for the bivariate two-sided correlation test of all variables in the same patient. The correlation coefficients are presented in a correlation matrix. Calculations showed that the current sample size was able to determine whether the correlation coefficient differs from zero if *r* ≥ 0.58 (*α* (two-tailed) ≤ 0.05, *β* = 0.20). The level of significance was set at *p* ≤ 0.05.

## Results

### Heel-rise endurance test and gait biomechanics

There were several significant correlations among parameters of the heel-rise endurance test and the gait variables (Table 2). More heel-rise repetitions correlated with higher speed (*r* = −0.52) and greater peak ankle power (*r* = 0.64), and with shorter stance phase (*r* = −0.52) and less peak ankle dorsiflexion angle (*r* = −0.64). Greater heel-rise height in cm correlated with higher speed (*r* = 0.55) and higher peak ankle power (*r* = 0.62), and with shorter stance phase (*r* = −0.76) and less peak ankle dorsiflexion angle (*r* = −0.49). Greater heel-rise total work correlated with higher speed (*r* = 0.57) and higher peak ankle power (*r* = 0.66), and with shorter stance phase (*r* = −0.63) and less peak ankle dorsiflexion angle (*r* = −0.62).

### Tendon length and gait biomechanics

Greater absolute ATRA correlated with longer stance time (*r* = 0.47) and greater peak ankle dorsiflexion angle (*r* = 0.48) (Table 2). There were no significant correlations related to absolute ultrasound measurement of tendon length and the gait variables.

**Table 2:** The correlations between gait variables and the heel-rise endurance test, ATRA and ultrasound measurement of tendon length

		Speed (m/s)	Stance (%)	Peak ankle dorsiflexion (°)	Peak ankle power (W/kg)
Heel-rise repetitions injured side (cm)	*r*	0.52	−0.52	**−0.64**	**0.64**
	*p* value	0.016	0.016	**0.002**	**0.002**
	ES	0.27	0.27	**0.41**	**0.41**
	*N*	21	21	**21**	**21**
Heel-rise height injured side (cm)	*r*	0.55	**−0.76**	−0.49	**0.62**
	*p* value	0.010	**0.000**	0.026	**0.002**
	ES	0.30	**0.57**	0.24	**0.40**
	*N*	21	**21**	21	**21**
Heel-rise total work (joule)	*r*	0.57	**−0.63**	**−0.62**	**0.66**
	*p* value	0.007	**0.002**	**0.003**	**0.001**
	ES	0.33	**0.39**	**0.39**	**0.44**
	*N*	21	**21**	**21**	**21**
Absolute ATRA (°)	*r*		0.47	0.48	
	*p* value	n.s.	0.030	0.028	n.s.
	ES		0.22	0.23	
	*N*		21	21	
Absolute UL (cm)	*r*				
	*p* value	n.s.	n.s.	n.s.	n.s.
	ES				
	*N*				

Bold text = *r* ≥ 0.58 (*α* (two-tailed) = 0.05, *β* = 0.20)

*CC* correlation coefficient, *ES* effect size (Cohen’s *d*, *r*^2^), *ATRA* Achilles tendon resting angle, *LSI* Limb Symmetry Index (injured/healthy side × 100), *n.s. * non-significant, *r* correlation coefficient, *UL* ultrasound

### Tendon length and calf muscle function

A greater absolute ATRA correlated with less heel-rise total work LSI (*r* = −0.44) and greater relative ATRA correlated with less heel-rise repetitions (*r* = −0.52) and less heel-rise total work LSI (*r* = −0.59) (Table 3). There were no significant correlations between the ultrasound measurement of tendon length and the calf endurance test.

**Table 3:** The correlations between ATRA, ultrasound measurement of tendon length and the heel-rise endurance test

		Heel-rise repetitions injured side (cm)	Heel-rise repetitions (LSI)	Heel-rise height injured side (cm)	Heel-rise height (LSI)	Heel-rise work injured side (joule)	Heel-rise work (LSI)
Absolute ATRA (°)	*R*						−0.44
	*p* value	n.s.	n.s.	n.s.	n.s.	n.s.	0.047
	ES						0.19
	*N*						21
Relative ATRA (°)	*R*		−0.52				**−0.59**
	*p* value	n.s.	0.016	n.s.	n.s.	n.s.	**0.005**
	ES		0.27				**0.35**
	*N*		21				**21**
Absolute UL (cm)	*R*						
	*p* value	n.s.	n.s.	n.s.	n.s.	n.s.	n.s.
	ES						
	*N*						
Relative UL (cm)	*R*						
	*p* value	n.s.	n.s.	n.s.	n.s.	n.s.	n.s.
	ES						
	*N*						

Bold text = *r* ≥ 0.58 (*α* (two-tailed) = 0.05, *β* = 0.20)

*CC* correlation coefficient, *ES* effect size (Cohen’s *d*, *r*^2^), *ATRA* Achilles tendon resting angle, *LSI* Limb Symmetry Index (injured/healthy side × 100), *n.s. * non-significant, *r* correlation coefficient, *UL* ultrasound

## Discussion

The most important finding of this study was that better performance in the heel-rise endurance test was moderately correlated with better ankle biomechanics during gait. The findings indicate that increased calf muscle endurance, especially the measure of heel-rise total work, is related to the ability to create greater force in the ankle joint in terms of functional activity such as gait. The heel-rise total work outcome combines heel-rise height and heel-rise endurance, and has previously been found to have an important clinical value since it has a greater ability to detect side-to-side differences, compared to with only measures of the number of repetitions performed [26]. Similar to healthy individuals, greater total work during the heel-rise endurance test has been found to correlate with higher plantar flexion moment and power during gait [31].

Peak ankle power was the gait variable strongest related to all parameters of the heel-rise endurance test. It can be considered as the most important of the included gait variables, since it is a sensitive measurement of force produced in the ankle joint, and, in turn, an indicator of gait speed and functional capacity. Moreover, all individual parameters of the heel-rise endurance test were related to a shorter stance phase relative to swing phase on the affected leg, which may suggest that better calf muscle endurance is related to less limping during gait.

There was a relationship between greater absolute ATRA and greater peak ankle dorsiflexion angle during gait. An elongated tendon may allow increased dorsal flexion in the ankle joint during stance before heel lift and toe-off. Previously, Manegold et al. [17] reported a correlation between greater tendon elongation, as measured with ultrasound, and greater peak dorsiflexion during level walking in patients after ATR on average 43.5 ± 12 months after surgical intervention. Furthermore, greater ATRA was correlated with inferior heel-rise reps LSI and heel-rise work LSI [17]. Zellers et al. [32] reported that increased relative ATRA, at 1 year or more after injury, was related to decreased heel-rise work LSI in patients both surgically and non-surgically treated patients after an acute ATR. The recovery of calf muscle endurance may be related to tendon elongation also in patients treated for CATR. In the study by Zellers et al. [32], greater relative ATRA was related to greater tendon length as measured with ultrasound, but in the present study, no relationship was found between the two tendon length measurements (Table 4 in Appendix). Surgical treatment with augmentation, as in patients with CATR, may complicate the ability to correctly identify the anatomical landmark used for the ultrasound tendon length measurement.

**Table 4:** An overview of all correlations between included variables

		Heel-rise repetitions injured side (cm)	Heel-rise repetitions (LSI)	Heel-rise height injured side (cm)	Heel-rise height (LSI)	Heel-rise work injured side (joule)	Heel-rise work (LSI)	Absolute ATRA (°)	Relative ATRA (°)	Absolute UL (cm)	Relative UL (cm)	Speed (m/s)	Stride length (m)	Stance (%)	Peak ankle dorsiflexion (°)	Peak ankle moment (Nm/kg)	Peak ankle power (W/kg)
Heel-rise repetitions injured side (cm)	CC		*.761*	*.803*	*.904*	*.945*	*.885*	−.432	−.421	.078	.037	*.518*	.231	−*.517*	−*.641*	*.488*	*.637*
	*p*-value		*.000*	*.000*	*.000*	*.000*	*.000*	.051	.057	.774	.892	*.016*	.313	*.016*	*.002*	*.025*	*.002*
	*N*		*21*	*21*	*21*	*21*	*21*	21	21	16	16	*21*	21	*21*	*21*	*21*	*21*
Heel-rise repetitions (LSI	CC	*.761*		*.65*	*.682*	*.689*	*.894*	−.379	−*.520*	−.251	.013	*.474*	.181	−*.576*	−*.732*	*.507*	*.540*
	*p*-value	*.000*		*.001*	*.001*	*.001*	*.000*	.090	*.016*	.349	.961	*.030*	.433	*.006*	*.000*	*.019*	*.011*
	*N*	*21*		*21*	*21*	*21*	*21*	21	*21*	16	16	*21*	21	*21*	*21*	*21*	*21*
Heel-rise height injured side (cm)	CC	*.803*	*.657*		*.839*	*.899*	*.713*	−.322	−.293	.057	−.194	*.548*	.494	−*.758*	−*.486*	*.582*	*.624*
	*p*-value	*.000*	*.001*		*.000*	*.000*	*.000*	.155	.198	.833	.471	*.010*	*.023*	*.000*	*.026*	*.006*	*.002*
	*N*	*21*	*21*		*21*	*21*	*21*	21	21	16	16	*21*	*21*	*21*	*21*	*21*	*21*
Heel-rise height (LSI)	CC	*.904*	*.682*	*.839*		*.888*	*.839*	−.384	−.392	−.159	−.247	*.516*	.312	−*.556*	−*.683*	*.438*	*.557*
	*p*-value	*.000*	*.001*	*.000*		*.000*	*.000*	.086	.079	.557	.356	*.017*	.168	*.009*	*.001*	*.047*	*.009*
	*N*	*21*	*21*	*21*		*21*	*21*	21	21	16	16	*21*	21	*21*	*21*	*21*	*21*
Heel-rise work injured side (joule)	CC	*.945*	*.689*	*.899*	*.888*		*.777*	−.429	−.327	.094	−.038	*.571*	.322	−*.626*	−*.621*	*.565*	*.660*
	*p*-value	*.000*	*.001*	*.000*	*.000*		*.000*	.052	.148	.729	.888	*.007*	.155	*.002*	*.003*	*.008*	*.001*
	*N*	*21*	*21*	*21*	*21*		*21*	21	21	16	16	*21*	21	*21*	*21*	*21*	*21*
Heel-rise work (LSI)	CC	*.885*	*.894*	*.713*	*.839*	*.777*		−*.438*	−*.593*	−.079	−.056	.430	.155	−*.556*	−*.704*	.396	*.549*
	*p*-value	*.000*	*.000*	*.000*	*.000*	*.000*		*.047*	*.005*	.770	.837	.052	.502	*.009*	*.000*	.075	*.010*
	*N*	*21*	*21*	*21*	*21*	*21*		*21*	*21*	16	16	21	21	*21*	*21*	21	*21*
Absolute ATRA (°)	CC	−.432	−.379	−.322	−.384	−.429	−.438^*^		*.555*	−.013	.144	.124	.096	*.473*	.479^*^	−.194	.145
	*p*-value	.051	.090	.155	.086	.052	*.047*		*.009*	.961	.595	.593	.678	*.030*	*.028*	.400	.531
	*N*	21	21	21	21	21	*21*		*21*	16	16	21	21	*21*	*21*	21	21
Relative ATRA (°)	CC	−.421	−*.520*	−.293	−.392	−.327	−.593^**^	*.555*		−.105	.330	−.094	−.039	.403	.403	.126	.063
	*p*-value	.057	*.016*	.198	.079	.148	*.005*	*.009*		.700	.212	.686	.865	.070	.070	.587	.786
	*N*	21	*21*	21	21	21	*21*	*21*		16	16	21	21	21	21	21	21
Absolute UL (cm)	CC	.078	−.251	.057	−.159	.094	−.079	−.013	−.105		.303	−.106	.330	−.029	.253	−.191	−.026
	*p*-value	.774	.349	.833	.557	.729	.770	.961	.700		.254	.696	.212	.914	.345	.478	.922
	*N*	16	16	16	16	16	16	16	16		16	16	16	16	16	16	16
Relative UL (cm)	CC	.037	.013	−.194	−.247	−.038	−.056	.144	.330	.303		.047	−.140	.368	.082	−.138	.126
	*p*-value	.892	.961	.471	.356	.888	.837	.595	.212	.254		.863	.606	.161	.762	.610	.641
	*N*	16	16	16	16	16	16	16	16	16		16	16	16	16	16	16
Speed (m/s)	CC	*.518*	.474	*.548*	*.516*	*.571*	.430	.124	−.094	−.106	.047		*.577*	−.400	−.381	.274	*.692*
	*p*-value	*.016*	.030	*.010*	*.017*	*.007*	.052	.593	.686	.696	.863		*.006*	.072	.089	.229	*.001*
	*N*	*21*	21	*21*	*21*	*21*	21	21	21	16	16		*21*	21	21	21	*21*
Stride length (m)	CC	.231	.181	*.494*	.312	.322	.155	.096	−.039	.330	−.140	*.577*		−.398	−.029	.220	.381
	*p*-value	.313	.433	*.023*	.168	.155	.502	.678	.865	.212	.606	*.006*		.074	.900	.339	.088
	*N*	21	21	*21*	21	21	21	21	21	16	16	*21*		21	21	21	21
Stance (%)	CC	−.517^*^	−*.576*	−*.758*	−*.556*	−*.626*	−*.556*	*.473*	.403	−.029	.368	−.400	−.398		*.525*	−*.519*	−.381
	*p*-value	*.016*	*.006*	*.000*	*.009*	*.002*	*.009*	*.030*	.070	.914	.161	.072	.074		*.015*	*.016*	.089
	*N*	*21*	*21*	*21*	*21*	*21*	*21*	*21*	21	16	16	21	21		*21*	*21*	21
Peak ankle dorsiflexion (°)	CC	−*.641*	−*.732*	−*.486*	−*.683*	−*.621*	*.704*	*.479*	.403	.253	.082	-.381	−.029	*.525*		−.397	−.351
	*p*-value	*.002*	*.000*	*.026*	*.001*	*.003*	*.000*	*.028*	.070	.345	.762	.089	.900	*.015*		.074	.119
	*N*	*21*	*21*	*21*	*21*	*21*	*21*	*21*	21	16	16	21	21	*21*		21	21
Peak ankle moment (Nm/kg)	CC	*.488*	*.507*	*.582*	*.438*	*.565*	.396	−.194	.126	−.191	−.138	.274	.220	−*.519*	−.397		*.660*
	*p*-value	*.025*	*.019*	*.006*	*.047*	*.008*	.075	.400	.587	.478	.610	.229	.339	*.016*	.074		*.001*
	*N*	*21*	*21*	*21*	*21*	*21*	21	21	21	16	16	21	21	*21*	21		*21*
Peak ankle power (W/kg)	CC	*.63*	*.540*	*.624*	*.557*	*.660*	*.549*	.145	.063	−.026	.126	*.692*	.381	−.381	−.351	*.660*	
	*p*-value	*.002*	*.011*	*.002*	*.009*	*.001*	*.010*	.531	.786	.922	.641	*.001*	.088	.089	.119	*.001*	
	*N*	*21*	*21*	*21*	*21*	*21*	*21*	21	21	16	16	*21*	21	21	21	*21*	

Correlation significant at the 0.05 level (two-tailed) are in italics; CC = correlation coefficient; ES = effect size (Cohen’s *d*, *r*^2^); ATRA = Achilles Tendon Resting Angle; LSI = Limb symmetry index (injured/healthy side × 100); r = correlation coefficient; UL = ultrasound

The fact that there are consistent moderate correlations between all parameters of the heel-rise endurance test and biomechanical gait variables, strengthens the credibility of a true relationship. The study’s primary limitation is the small cohort, which affects the solidity of the correlations with lower correlation coefficients (*r* ≤ 0.58). Moreover, a correlation analysis only assesses the relationship between the variables and does infer any causality. The included patients are heterogeneous in terms of age and time between injury and surgery. However, since they received similar surgical treatment and rehabilitation, and the fact that large individual variations are common in patients with CATR, the findings are still considered to have external validity. The findings indicate that maximal recovery of calf muscle endurance is of importance for functional recovery after surgical treatment of CATR.

## Conclusion

Better calf muscle endurance is moderately correlated to greater ankle biomechanics during gait in patients surgically treated for CATR. Heel-rise total work was the functional parameter most strongly related to several aspects of gait. The findings indicate that heel-rise endurance may be a clinical proxy for power development in the ankle joint during gait.

## Appendix

See Table 4.

## Abbreviations

ATR: Achilles tendon rupture
ATRA: Achilles tendon resting angle
BMI: Body mass index
CATR: Chronic Achilles tendon rupture
LSI: Limb Symmetry Index
OTJ: Osteotendinous junction
MTJ: Musculotendinous junction
